# 

**DOI:** 10.1038/sj.bjc.6600379

**Published:** 2002-01-02


expression was conducted at the protein level to attempt to explain the differential responsiveness of these cell lines to IL-8. IP-Western analysis revealed that CXCR1 expression is higher than that of CXCR2 in both cell lines. Flow cytometry and immunocytochemistry demonstrated detectable CXCR1 and CXCR2 cell surface receptor expression in PC3 cells, but also revealed that CXCR1 and CXCR2 are predominantly localised to distinct foci within the cytoplasm of PC3 cells and are almost exclusively localised to intracellular compartments of the PNT1-A cells. ELISA analysis has revealed that PNT1A cells exhibit a 5-7 fold increase in IL-8 secretion relative to PC3 cells suggesting that the poor growth response of PNT1A cells to IL-8 may reflect their elevated IL-8 production and thus the marked desensitisation of the receptors. Our work to date has demonstrated all of the essential features of an autocrine IL-8 loop in these prostate cell lines: IL-8 secretion, expression and localisation of the IL-8 receptors and in the case of PC3 cells, a growth response to the agonist. Ongoing experiments are focused on blocking the signalling capacity of endogenous and exogenous IL-8 via CXCR1 and CXCR2 using specific antibodies, to identify which IL-8 receptor is coupled to the growth-promoting pathway.

**P197**

**MELANOMA CELL ATTACHMENT, INVASION AND INTEGRIN EXPRESSION IS UPREGULATED BY THE PROINFLAMMATORY CYTOKINE TNF- AND SUPPRESSED BY THE ANTI-INFLAMMATORY PEPTIDE -MSH.**

*E. Katerinaki[1,4], N. Zhu[2], J.W. Haycock[2], P. Eves[1], G.S. Evans[3], P.C. Lorigan[4] and S. MacNeil[1,2].
[1]University of Sheffield, Clinical Sciences Centre, Northern General Hospital, Sheffield, S5 7AU, [2]Department of Engineering Materials, University of Sheffield, Sheffield, S1 3JD, [3]Child Health University Division of Clinical Sciences, Stephenson Unit, The Children's Hospital, Sheffield, S10 2TH, [4]Academic Department of Clinical Oncology, Weston Park Hospital, Sheffield, S10 2SJ.

Aim: Do proinflammatory cytokines involved in wound healing promote melanoma invasion? This is important from both a biological and a clinical point of view as they can potentially affect local tumour recurrence. The purpose of this study was to examine *in vitro* the effects of the proinflammatory cytokine TNF- and the anti-inflammatory peptide -MSH on attachment, invasion and integrin expression in a human melanoma cell line.

Materials and methods: The human cutaneous melanoma cell line HBL, human recombinant TNF- and -MSH were used to examine the effects of TNF- and -MSH on cell attachment (at 24 h), invasion through fibronectin coated micropore membranes (20 h) and integrin expression (detected by Western immunoblotting) measured at 24 h.

Results: 24 h exposure to TNF- enhanced cell attachment to tissue culture plastic, collagen I and collagen IV (table shows the results of tissue culture plastic), increased the invasion of melanoma cells through fibronectin and upregulated the expression of integrin subunits $_3$, $_4$ and $_1$. Exposure of cells to -MSH significantly reduced cell attachment and invasion and slightly reduced integrin expression. The simultaneous exposure of cells to -MSH and TNF- clearly attenuated the response of cells to TNF- with respect to invasion and expression of $_3$ and $_4$ integrin subunits.

| | TNF- (300 U/ml) | -MSH ($10^{-7}$ M) | TNF- (300 U/ml) and -MSH ($10^{-7}$ M) |
|---|---|---|---|
| Attachment | +10% | -23% | To be done |
| Invasion | +45% | -47% | -16% |
| Integrin expression | | | |
| $_3$ | +28% | -15% | -18% |
| $_4$ | +90% | -15% | -20% |
| $_1$ | +65% | -22% | +25% |

Conclusions: The ability of TNF- to increase integrin subunit expression, cell attachment and cell invasion is consistent with an inflammatory environment promoting melanoma invasion; the ability of -MSH to attenuate the response to TNF- is also consistent with -MSH acting in an anti-inflammatory capacity to oppose metastatic spread.

**P198**

**INTERLEUKIN-12 POLYMORPHISMS MAY INFLUENCE THE HOST MUCOSAL RESPONSE TO HELICOBACTER PYLORI.**

EJ Dickson*, AJ Morris[+], C Craig, J Eskdale, G Gallagher, RC Stuart.
University Dept of Surgery and Dept of Medicine[+], Glasgow Royal Infirmary.

**AIM:** Cytokine function is believed to play a role in benign and malignant mucosal disease. Cytokine polymorphisms at host genetic level may be important in determining susceptibility to different mucosal responses. The aim of this study was to examine the polymorphisms of interleukin-12 (IL-12), a pro-inflammatory cytokine, in a large benign dyspeptic group with a high prevalence of H. pylori infection.

**METHODS:** The study cohort, a dyspeptic population (n=300) on acid suppression therapy, was subdivided into ulcer (n=96), non-ulcer (156) and "not investigated" (n=48) categories. DNA was extracted from peripheral blood leucocytes and the distribution of the IL-12 alleles was determined by polymerised chain reaction, followed by restriction enzyme digestion.

**RESULTS:** The genotype frequencies for the study population were A/A 66.3%, A/C 29.7% and C/C 4%, which are very similar to our normal control frequencies of 64.2%, 31.6% and 4.2% respectively. However, the genotype frequencies for the ulcer (42 patients) versus non-ulcer (56 patients) in the helicobacter positive group were 59.5%, 38.1%, 2.4% and 78.6%, 16.1%, 5.4% respectively (p=0.042).

**CONCLUSIONS:** There is a significant difference between the helicobacter positive ulcer and non-ulcer patients. We previously demonstrated a similar increased frequency of the A/A genotype in H. pylori positive gastro-oesophageal cancer patients. This study therefore suggests that the polymorphisms of the pro-inflammatory cytokine IL-12 may influence the host mucosal response to H. pylori infection.

**P199**

**CHARACTERISATION OF THE GENE ENCODING EMAP-II, A TUMOUR-DERIVED PRO-INFLAMMATORY CYTOKINE**

YM Heng*, P Symonds and JC Murray. Cancer Research UK Dept of Clinical Oncology, University of Nottingham

The 20kDa pro-inflammatory cytokine EMAP-II, secreted by tumour cells under stress, is synthesized as a precursor of 34kDa (proEMAP-II) and was originally believed to be encoded by a 1.1kbp cDNA. However, the origin of EMAP-II remains unclear. Recently, a 1.25kbp cDNA reported to encode p43, a 43kDa component of the hamster multisynthase complex, was found to have high homology with the proEMAP-II cDNA, but contained additional 5' sequence. We have used a variety of genomic and RNA-based techniques to help elucidate the relationship between EMAP-II and p43 proteins. By database searches we have identified a genomic contig of ~117kbp containing the functional EMAP-II gene. This gene is approximately 30kbp in size, comprises 7 exons, and possibly shares a bi-directional promoter with a novel gene, arranged in head-to-head fashion. By RT-PCR, we cloned additional 5' cDNA sequences from human cells, corresponding to the first 170bp of sequence of the hamster p43 cDNA. The two species demonstrate sequence divergence in the 5' region of the cDNA. The hamster sequence contains translation start sites appropriate for the predicted p43 protein as well as 34kDa EMAP-II proteins, while the human sequence contains a start site for a 34kDa protein but not p43. This is in agreement with western blotting with polyclonal antibodies against EMAP-II, which revealed the presence of ~43kDa and 34kDa bands in extracts of hamster cells. Human cell extracts however, do not express a 43kDa band, but only a 34kDa band. Thus it appears that humans do not express a homologue of the hamster p43 protein. The reasons for this evolutionary divergence are currently unclear. We have also explored the expression of EMAP-II mRNA by northern analysis to determine the size and distribution of the mRNA in human tissues. EMAP-II mRNA was most abundant in the adult testis. Furthermore, the same EMAP-II probe identified 3 RNA transcripts of sizes ~1.3kb, 2.0kb and 2.6kb. A blot of mRNA extracted from several tumour cell lines also showed expression of the same 3 transcripts as in normal tissues. The significance of multiple hybridising transcripts is unknown, but these could represent alternatively or incompletely spliced transcripts. We are currently investigating this and the activity of the promoter region by reporter assays.

*This work is supported by Cancer Research UK*

© 2002 Cancer Research UK

### P200

### TUMOUR-DERIVED EMAP-II INHIBITS PROLIFERATION AND INDUCES APOPTOSIS IN LYMPHOCYTES

K Rice*, P Symonds, YM Heng, W Ward, I Todd[1], RA Robbins[1] and JC Murray. Cancer Research UK Dept of Clinical Oncology, City Hospital, and [1]Division of Immunology, Queens Medical Centre, University of Nottingham

EMAP-II (endothelial monocyte-activating polypeptide II) was first detected in supernatants of murine fibrosarcoma cells. *In vitro,* EMAP-II induces procoagulant activity on the surface of endothelial cells, increased expression of E-and P-selectins and TNF Receptor-1, and is chemotactic for monocytes and neutrophils. More recent data suggest that EMAP-II induces apoptosis in a number of cell types. Therefore EMAP-II may play a role in tissue remodeling, by inducing cell death in selected populations, simultaneously encouraging the activity of phagocytic cells to clear cellular debris. In spite of this accumulating *in vitro* data, the role of EMAP-II in tumours is not understood. We have hypothesized that EMAP-II may regulate activity of immune effector cells within neoplastic tissues, and have investigated its effects on lymphocytes. In a first set of experiments, we treated peripheral blood mononuclear cell preparations (PBMC) with recombinant EMAP-II, prior to stimulation with PHA to induce lymphocyte proliferation. EMAP-II caused a dose-dependent inhibition of $^3$H-thymidine incorporation (70% suppression at 200nM). Consistent with this, use of the CFSE dye dilution technique demonstrated a significant reduction in the number of cell divisions following EMAP-II treatment. Apoptosis was assessed in the same experiments by flow cytometry using the Annexin-V/propidium iodide technique. Treatment with EMAP-II induced apoptosis in lymphocytes stimulated by mitogen PHA, but not in un-stimulated cells. In the second series of experiments, DLD-1 colorectal adenocarcinoma cells, which express EMAP-II, were co-cultured with Jurkat (T-cell leukaemia) cells, to determine whether EMAP-II produced by the colorectal tumour cells can induce cell death in Jurkat cells. DLD-1 colorectal cancer cells express the 34kDa form of EMAP-II; and we showed by ELISA that these cells secrete EMAP-II into the culture medium in response to the combination of TNF-alpha and Interferon-gamma (TNF/IFN). By Annexin-V/PI labeling, treatment of Jurkat cells with recombinant EMAP-II alone induced significant apoptosis. Co-culture of Jurkat cells with untreated DLD-1 cells induced some apoptosis of Jurkat cells; however a significant increase in killing of Jurkat cells was observed following pre-treatment of DLD-1 cells with TNF/IFN. Similar results were obtained when Jurkat cells were incubated with conditioned medium from TNF/IFN-treated DLD-1 cells. These effects could be partially reversed by addition of polyclonal antibodies against EMAP-II to the co-culture. Our data suggest that EMAP-II released by tumour cells may be cytotoxic to lymphocytes through an unknown mechanism, and may constitute a component of a novel immunosuppressive pathway in solid tumours.

*This work is supported by Cancer Research UK*

### P201

### VEGF IS A POTENT ANGIOGENIC FACTOR IN TUMOURS OF THE EWING'S SARCOMA FAMILY

*[1]Surita Dalal , [2]Gerald McMahon and [1]Susan A Burchill
[1]Candlelighter's Children's Cancer Research Laboratory, Cancer Research UK Clinical Centre, St James's University Hospital, Leeds, LS9 7TF, UK and [2]SUGEN Inc, California, USA

Angiogenesis plays an important role in tumour growth, invasion and metastasis. Recently we have demonstrated that enhanced neovascularisation is a marker of poor prognosis for over-all and event-free survival in patients with tumours of the Ewing's Sarcoma Family (ESFTs). This suggests that the process of angiogenesis plays an important role in these tumours. The aims of this study were to investigate the expression and production of the angiogenic growth factors vascular endothelial growth factor (VEGF), basic fibroblast growth factor (bFGF) and platelet derived growth factor (PDGF) in ESFT cells. Potential autocrine and/or paracrine effects of these growth factors were also investigated. The effects of anti-tyrosine kinase receptor compounds targeting VEGF (SU5416) or VEGF, bFGF and PDGF (SU6668) receptors on ESFT growth *in vitro* and *in vivo* were evaluated.

Production and secretion of VEGF, bFGF, PDGF-AB and PDGF-BB by 6 ESFT cell-lines was examined by reverse transcriptase polymerase chain reaction (RT-PCR) and ELISA (R+D Systems). Expression of VEGF and its receptors Flt-1 and Flk-1 (KDR) was investigated by RT-PCR and Western blotting (WB). Potential autocrine and/or paracrine effects of VEGF were investigated using an antihuman VEGF neutralising antibody (R+D Systems) and the anti-tyrosine kinase receptor molecules (SUGEN). The effects of the anti-tyrosine kinase receptor compounds SU6668 and SU5416 (SUGEN) were also investigated *in vivo* following ESFT implantation in NuNu mice.

All 6 ESFT cell lines studied produced and secreted VEGF into the media, as demonstrated by RT-PCR of cell extracts and ELISA on cell-conditioned media. In contrast bFGF, PDGF-AB and PDGF-BB were not detected in conditioned media. Different isoforms of VEGF were identified by WB; these were confirmed by RT-PCR to be VEGF121 and 165. ESFT cells differentially expressed Flt-1 and Flk-1 (KDR), although using neutralising antibodies to VEGF or anti-tyrosine kinase receptor compounds no evidence of an autocrine and/or paracrine role for VEGF was found. Treatment of NuNu mice ip with SU5416 (25mg/kg/day) or SU6668 (100mg/kg/day) significantly reduced ESFT xenograft growth ($p<0.05$, $p<0.001$ respectively). Furthermore SU6668 (100mg/kg/day) caused a regression of established ESFT xenografts.

In conclusion, VEGF appears to be an important angiogenic growth factor in ESFT. Targeting VEGF and/or VEGF receptors may be of therapeutic benefit in patients with ESFT.

### P202

### MVD PREDICTS DISEASE-FREE AND OVERALL SURVIVAL IN TUMOURS OF THE EWING'S SARCOMA FAMILY (ESFT)

Andrea Simpson[1]*, Robert Grimer[2], Charles Mangham[3], Catherine Cullinane[4], Ian Lewis[5], Susan A Burchill[1]. [1]Candlelighter's Children's Cancer Research Laboratory, Cancer Research UK Clinical Centre, [4]Department of Pathology and [5]Paediatric Oncology, St James's University Hospital, Leeds, LS9 7TF, [2]Royal Orthopaedic Hospital, Birmingham B31 2AP, [3]Department of Musculoskeletal Pathology, Birmingham B29 6AY, UK.

There is a need for new prognostic markers and therapeutic targets in ESFTs. Since angiogenesis plays an important role in the growth, invasion and metastasis of many solid tumours, we have evaluated the prognostic importance of angiogenesis in ESFTs.

Micro-vessel density (MVD), an established marker of angiogenesis, was assessed in cryostat sections of 29 primary ESFTs by immunohistochemistry for the endothelial cell marker CD31 (Pecam-1; Dako). Bound antibody was detected using a biotin-conjugated rabbit anti-mouse IgG (Dako) and an avidin-biotin-peroxidase complex (Dako). The brown precipitate was developed using 3',3' diaminobenzidine (DAB) substrate and sections counterstained with haematoxylin. Each section was scanned on a Zeiss Axioplan microscope at 160x magnification to identify three areas with greatest micro-vessel density, the vessel count was performed at 250x magnification for an overall area of 0.79mm$^2$ for each 'hotspot' identified. The mean micro-vessel count was calculated for each section and MVD expressed as the number of micro-vessels per mm$^2$. The tumours were classified into two groups, negative/low MVD (MVD<100) and high MVD (MVD>100). The time to first event and current disease status for each patient was obtained, and Kaplan-Meier survival curves plotted. Statistical differences between the curves were compared using the Log-Rank test. Tumour samples and clinical outcome information were only obtained when informed consent had been given.

Twenty percent (7/29) of ESFTs had high MVD, consistent with their haematogenous ESFT macroscopy. Mean time to first event in patients with these tumours was 17 months, and overall survival 28 months. In contrast in the remaining 76% (22/29) time to first event was 32 months and over all survival was 48 months. High MVD was a statistically significant marker of both poor overall ($P=0.04$) and event free ($P= 0.01$) survival in this small cohort of patients.

In conclusion, MVD is a marker of poor prognosis in patients with ESFT and might therefore be used to stratify patients with ESFT for therapy. Since MVD is prognostically significant for disease-free and overall survival this suggests that angiogenesis may play an important role in ESFT progression and metastasis. This implies that the process of blood vessel formation may be a potential target for therapeutic intervention in patients with ESFT.

© 2002 Cancer Research UK

**P203**

**EFFECTS OF SIGNALLING INHIBITORS ON ENDOTHELIAL CELL FUNCTIONS REQUIRED FOR NEOANGIOGENESIS.**

S. Brader*, W. Court, G. Box, S.A. Eccles, P. Workman, Cancer Research UK Centre for Cancer Therapeutics, Institute of Cancer Research, Sutton, Surrey, SM2 5NG, UK.

Neoangiogenesis is critical to tumour growth and metastasis, and hence is an important generic target for therapeutic intervention in cancer. Angiogenesis is driven by a variety of cytokines; the most important of which are the vascular endothelial growth factor (VEGF) family. Several agents are in clinical development which attempt to prevent neoangiogenesis via inhibition of VEGF receptor activation (e.g. SU5416) or binding of endothelial integrins to extracellular matrix (eg Vitaxin) and tubulin assembly (e.g Combretastatin A4). Signalling pathways in endothelial cells are generally poorly understood, but we aim to explore selected targets for which there is some evidence for a role in tumour angiogenesis. The studies reported here have explored inhibitors of phosphoinositide 3 kinase (PI3K) and phospholipase C (PLC) in relation to endothelial cell (EC) functions required for neoangiogenesis, *viz* proliferation, chemotaxis, matrix proteolysis and tubule differentiation.

A relationship has been shown between PI3K activity and survival/proliferation and migration in several types of cells. We found that the PI3K inhibitor, LY294002, decreased human EC proliferation ($IC_{50}$ = 3μM) and migration though a 3μM Transwell filter ($IC_{50}$ = 1.5μM). It also reduced production of matrix metalloproteinase 2 (MMP-2) ($IC_{50}$ =3μM) in response to VEGF, as shown by gelatin zymography and confirmed by ELISA.

PLCγ has also been shown to be important in tumour cell motility and invasion. U73122, a PLC inhibitor, inhibited endothelial cell proliferation at 6.5μM, migration ($IC_{50}$ = 0.1μM) and MMP-2 production ($IC_{50}$ = 3μM). Also, in contrast to LY294002, U73122 decreased EC differentiation in the matrigel tube formation assay, totally abolishing tube formation at 3μM. The inactive analogue U73343, had no significant effects on EC functions. Interestingly, we found that EC grown on “physiological” substrates such as collagen 1 matrix were less sensitive to PI3K and PLC inhibitors, suggesting care in interpretation of effects seen in standard 2D plastic culture systems.

These results identify PI3K and PLC as important molecules in endothelial cell responses to VEGF. Current inhibitors are of relatively low specificity, and are not ideal for use *in vivo*. Future generations of PI3K and PLC inhibitors may have a useful role in cancer therapy by inhibition of several steps in the angiogenic cascade.

**P204**

**INFLUENCE OF HIF-1 EXPRESSION ON TUMOUR GROWTH, METASTATIC POTENTIAL AND RESPONSE TO THERAPY IN EXPERIMENTAL TUMOUR MODELS**

***Sarah J. Lunt*, Kaye J. Williams, Ian J. Stratford***
School of Pharmacy & Pharmaceutical Sciences, University of Manchester, Oxford Road, Manchester UK M13 9PL

Tumour hypoxia is an adverse prognostic indicator for local control by radiotherapy, malignant progression and metastatic potential. The incidence of metastases has not only been linked to hypoxia itself but to the expression of the transcription factor HIF-1 (Hypoxia Inducible Factor-1). In order to uncouple the contribution of abnormal physiology (hypoxia) and de-regulated expression of HIF-1 in tumour growth metastases growth and response to therapy, we have used a panel of syngeneic and experimental tumours growing in mice. The panel includes Hepa-1 wt and C4 cells which are proficient and deficient in HIF-1 function respectively. Both cell types form tumours in nude mice and equal sizes have similar levels of hypoxia. However, the C4 tumours are formed more slowly and are much more responsive to ionising radiation than are wt tumours. This is due to hypoxic cells not contributing to tumour radiation resistance. Interestingly, administration of Hepa-1 wt and C4 cells to mice via tail vein injection results in a similar efficiency of lung colony (artificial metastasis) formation. A second pair of cell types are the rat glioblastoma lines CNS-1 and 9L. CNS-1 cells have a robust HIF-1 response, (16 fold induction of HRE-driven marker gene expression under hypoxic conditions) whereas the 9L cells show no hypoxia mediated gene induction but rather constitutive over expression of HIF-1 function in air (170 fold). It is of note, the 9L cells when grown as tumours in vivo respond to radiation in a similar manner to the Hepa-1 C4 cells (ie. hypoxic cells not contributing to radiation resistance).

The last group of tumours include B16 f10 cells syngeneic in C57 mice and KHT cells syngeneic in C3H mice. The B16 cells were chosen in this study because they have the greatest HIF-1 response (300 fold due to little, if any, aerobic HIF expression). The KHT cells were used because they have an extremely reproducible temporal and special distribution of metastasis from a primary subcutaneous tumour. Taken together, this panel of tumours will allow us to dissect out the importance of hypoxia, hypoxia mediated gene expression and HIF-1 function in tumour growth, metastatic potential and response to therapy.

**P205**

**STUDYING THE ROLE OF iNOS IN TUMORIGENESIS UTILIZING THE ECDYSONE SYSTEM**

Rana S. Al-Assah *, Rachel L. Cowen, Edwin Chinje, Ian Stratford
Experimental Oncology Group, Department of Pharmacy, University of Manchester, UK

Studies have shown that nitric oxide generated by the enzyme nitric oxide synthase (iNOS) can have pro-tumor effects when produced in low levels and anti-tumor effects when produced in high levels. (Edwin C. Chinje and Ian J. Stratford –1997-Essays in Biochemistry **32**: 61) Since stable clones that constitutively express very high levels of NO are often difficult to grow because of NO-induced toxicity, we have chosen an inducible approach. The ecdysone system was used particularly because of its low basal level and high fold induction. After transfecting the cells with the receptor vector (pVgRXR) and the response vector (pIND *SP*1) (which has a multiple cloning site to insert the gene of interest *lac*Z or iNOS), gene expression is induced by the addition of the steroid ecdysone or its analogue ponasterone A, (Pon A). (Enrique Saez, *et al.* –1997- Current Opinion in Biotechnology **8**: 608) (Enrique Saez, *et al.* –2000- PNAS **97**: (26) 14512). Since there is no data about the level at which the effect of NO turns from pro- to anti-tumorigenic, the aim of this study is to tightly regulate iNOS gene expression and consequently NO production so that the level can be resolved. This will then allow the interpretation of the dual role of NO on apoptosis and angiogenesis.

The ecdysone system was initially evaluated using the *lac*Z reporter gene. A stable clone of HT-1080 (human fibrosarcoma) expressing the ecdysone responsive cassette was generated. *In vitro*, the *lac*Z gene expression and activity as a function of Pon A concentration (0 to 20μM for 24 hours of Pon A exposure) was observed. Results showed increasing activity of the *β*-galactosidase enzyme with maximum induction of 17 fold at 20μM. The stable clone was then grown *in vivo* to a size of 250-300 $mm^3$ and Pon A administered by intraperitoneal injection (0, 1, 2 mg/mouse). After 24hrs the tumors were excised. They were either fixed or treated with X-gal staining or homogenized and *β*-galactosidase activity measured. The results showed increasing activity as the concentration of Ponasterone A increased.

Having tested the ecdysone system using the *lac*Z reporter gene, the system was used to tightly regulate iNOS levels. A stable clone of HT-1080 containing the iNOS gene inserted in the ecdysone cassette was generated. The clone was exposed to a range of Pon A concentrations (0 to 20 μM) and assayed to measure iNOS activity. The nitrite/nitrate content measured in the plasma reflected an increase in iNOS expression as Ponasterone A concentration increased. An immunocytochemistry approach was also used to measure iNOS induction. After staining the cells with iNOS specific antibodies, it was found that the percentage of immunoreactive cells increased with increasing Pon A concentration reaching a maximum of 21% with 30μM Pon A. The NOS L-Citrulline assay showed that the clone exhibited maximal iNOS activity levels (21.5mg/ml/min) when exposed to 20μM Pon A as compared with no activity with the wild type HT-1080. These results provide us with a unique opportunity to study the dual role of NO on apoptosis, tumor growth, and vascularization.

© 2002 Cancer Research UK

**P206**

**EVALUATION OF DOMINANT NEGATIVE AND SMALL MOLECULAR INHIBITORS OF HYPOXIA MEDIATED TRANSCRIPTION FOR POTENTIAL THERAPEUTIC APPLICATION**

L.M. Brown*[1], K.J. Williams[1], R.L. Cowen[1], G. Melillo[2], E. Sausville[2], I.J. Stratford[1] School of Pharmacy and Pharmaceutical Sciences, University of Manchester, Manchester, M13 9PL[1]; Tumour hypoxia laboratory, DTP, NCI, NIH, MD, U.S.A.[2]

Hypoxic regions in tumours develop because tumour cell growth is faster than the growth of the endothelial cells that compose the blood vessels and also because the newly formed vascular supply is disorganised. In order for tumour cells to survive and proliferate under these conditions they initiate the transcription of a number of genes. These include glycolytic enymes e.g. phosphoglycerate kinase (PGK), angiogenic factors e.g. vascular endothelial growth factor, regulators of blood flow e.g. nitric oxide synthase and regulators of red blood cell production e.g. erythropoietin. We have previously demonstrated that cell lines that lack subunits of the best-characterised transcription factor responsible for hypoxic induction of genes (HIF-1) are less tumourigenic than wildtype cells. In addition, HIF-1 over expression is common in malignant disease and has recently been identified as an adverse prognostic indicator in a variety of cancers. Hence, this transcription factor could be a valid therapeutic target. There are two main transcription factors that facilitate hypoxic control of gene expression, hypoxia inducible factor 1 (HIF-1) and hypoxia inducible factor 2 (HIF-2). They are heterodimers. Both transcription factors share the same $\beta$ subunit that is stable in normoxic conditions. The $\alpha$ subunits share 48% sequence identity and are subject to oxygen dependent degradation. In this study we have investigated the consequences of producing dominant negative forms of the $\alpha$ subunits by removing domains essential to their function but that should still allow heterodimers to form with the $\beta$ subunit. The functionality of the dominant negatives has been tested *in vitro* using transient co transfection of a vector containing a hypoxic response element (HRE) driven luciferase reporter and a vector expressing the dominant negative $\alpha$ subunit. The dominant negative $\alpha$ subunits reduced PGK, lactate dehydrogenase A (LDH-A) and carbonic anhydrase 9 (CA-9) HRE driven luciferase reporter gene expression in breast carcinoma, glioma, fibrosarcoma and lung carcinoma cell lines under hypoxic conditions. Along side the dominant negative study a small molecular inhibitor is also being validated using the same reporter techniques and has shown robust inhibition of HRE-mediated transcription. Stable cell lines are now being generated that over express the dominant negative proteins. This will allow the effect of inhibition of hypoxia induced gene expression on cell growth *in vitro* and *in vivo* to be investigated. In addition these constructs are being incorporated into an adenoviral system to evaluate the potential therapeutic effects of this dominant negative approach on established tumour xenografts.

**P207**

**TUMOUR NECROSIS CORRELATES WITH ANGIOGENESIS AND IS A PROGNOSTIC FACTOR IN MALIGNANT MESOTHELIOMA**

JG Edwards[1], D Swinson[1]*, JL Jones[2], G Cox[1], KJ O'Byrne[1]
[1]Department of Oncology, University of Leicester, Osborne Building, Leicester Royal Infirmary, and [2]Breast Cancer Research Unit, Department of Pathology, University of Leicester, Leicester, UK

**Aims**

Malignant mesothelioma (MM) is a fatal tumour of the pleura, associated with asbestos exposure, which is expected to double in incidence by 2020. MM is resistant to current therapy and has a median prognosis of approximately 6 months. Angiogenesis, as assessed by microvessel counts (MVCs) is an established prognostic factor in MM. Microscopic tumour necrosis (TN) has not been described or characterised in MM, although necrosis is a poor prognostic factor in other solid tumours. The aim of this study was to evaluate the incidence, correlations and significance of TN in MM.

**Methods**

Clinicopathological prognostic factors and European Organisation for Research and Treatment of Cancer (EORTC) and Cancer and Leukemia Group B (CALGB) prognostic scores were derived from case-note review of a cohort of 171 MM patients presenting between 1987 and 2001. MVCs were obtained from CD34-immunostained tumour sections. TN was assessed in routine formalin-fixed, paraffin-embedded, Haemotoxylin and Eosin stained tumour sections by two independent observers (blind to outcome) with a scale 0 (no necrosis) to 3 (frequent/large areas of necrosis). TN was correlated with survival by Kaplan-Meier and Log Rank analysis. A stepwise, multivariate Cox model was used to evaluate the impact on survival of TN, MVCs, known prognostic factors and the prognostic scoring systems proposed by the EORTC and CALGB. Patients surviving less than 30 days were excluded to avoid bias from peri-operative deaths.

**Results**

171 patients were assessed, of whom 17 (10%) died within 30 days. Overall median survival was 186 days. TN was identified in 39 cases (23%). TN was correlated with a high preoperative platelet count (p=0.04), and low haemoglobin (p=0.01) and high MVC (p=004). The presence of TN was associated with poor survival in univariate Cox proportional hazards analysis (HR 1.74, 95%CIs 1.15 – 2.63, p=0.009). Median survival in patients with and without TN was 158 and 248 days respectively. TN contributed to both CALGB (p=0.02) and EORTC (p=0.02) but not to MVCs (p=0.71) in multivariate Cox models.

**Conclusions**

TN necrosis correlates with angiogenesis in MM. Assessment of TN is a quick and reproducible technique, providing prognostic data in MM which is independent of the CALGB and EORTC prognostic scoring systems.

**P208**

**ENDOSTATIN IN CIRCULATING BLOOD CELLS**

Nash GF*, Chopada A, Kakkar AK, Imperial College, London

INTRODUCTION

Endostatin is an endogenous angiogenesis inhibitor demonstrated to reduce tumour growth *in vivo*, however it has few *in vitro* effects and little is known about either its source or mechanism of action. Vascular endothelial growth factor (VEGF), an important angiogenic stimulant opposing the effects of endostatin, is released by platelets and neutrophils. We aimed to investigate whether platelets or neutrophils also store endostatin.

METHODS

We measured the effect on platelets of endostatin addition, using a standard platelet aggregometer. In addition, endostatin release was measured by standard ELISA using pooled platelets obtained from the national blood bank. Washed platelets were compared to platelet-rich plasma (PRP), both at $1x10^8$/ml concentration. After ethical consent, blood was collected from 21 controls and 21 units of bank blood and after centrifugation at 3000 rpm the supernatants underwent endostatin ELISA.

RESULTS

Recombinant endostatin added to washed platelets demonstrated impressive inhibition of thrombin-induced aggregation, though this effect was wholly attributable to the endostatin buffer. There was a threefold increase in endostatin on stimulating pooled PRP (20.6 ng/ml vs. 60.4 ng/ml, P<0.001) with one unit of thrombin. Washed platelets released 1.5ng/ml (+/- 0.4) endostatin compared to washed platelet lysate 4.4 ng/ml (+/- 1.6, P<0.001). Clinically endostatin levels were not associated with platelet counts, but were positively correlated with neutrophil counts (R= 0.42, P=0.05), this was further supported by the finding that the total endostatin levels in the buffy coat (white cells) of donor blood was 20.0 ng/ml (+/- 0.88).

CONCLUSIONS

These data suggest that rather than endostatin inhibiting platelets, human platelets release endostatin upon thrombin stimulation. This together with the finding that endostatin levels correlate with neutrophil count strongly suggest that the manipulation of platelet and neutrophil activation may have important effects upon the endostatin levels of cancer patients.

**P209**

**CYCLOOYGENASE INHIBITORS MODULATE ENDOTHELIAL CELL APOPTOSIS, PROLIFERATION AND ANGIOGENESIS**

[#] SH Teh*, ADK Hill, A Lee, L Young, E McDermott, N O'Higgins. Depart. of Surgery, St Vincent's University Hospital, Ireland. Depart. of Surgery[#], Mayo Clinic, USA

**Aims: -** Several mitogens, including basic fibroblast growth factor (bFGF), are secreted during tumour growth. This pro-angiogenic environment

© 2002 Cancer Research UK 

enhances the process of tumour angiogenesis. We investigate the interaction of bFGF and COX inhibitors on endothelial cells apoptosis, proliferation and tubal formation. **Methods and Results: -**bFGF exerts potent cytoprotective (by intracellular nucleosomes assay and TUNEL) and pro-proliferative effect (by BrdU incorporation assay) on human umbilical vein endothelial cells. Non-selective COX inhibitor (aspirin) and selective COX II inhibitor (meloxicam) but not selective COX I inhibitor (SC 560) are pro-apoptotic and anti-proliferative in quiescent and bFGF stimulated endothelial cells. bFGF increased expression of prostaglandin $E_2$, a COX II cleaved arachidonic acid product. Both bFGF and $PGE_2$ induced a significant increase in tubal formation. Aspirin and meloxicam prevented neovascularization of endothelial cells even in the presence of bFGF. **Conclusion: -** Overall our data demonstrate that bFGF and $PGE_2$ are potent pro-angiogenic factors. COX inhibitors can negatively modulate the angiogenic effect of bFGF. It also inhibits the activities of COX enzyme and decreases production of $PGE_2$ which subsequently leads to inhibition of the process of angiogenesis.

**P210**

**THE MECHANISM OF ACTION OF ENDOSTATIN ON QUIESCENT AND STIMULATED ENDOTHELIAL CELLS**

#SH Teh*, ADK Hill, A Lee, McDermott E, O'Higgins N.
Depart. of Surgery, St Vincent's University Hospital, Ireland. Depart. of Surgery #, Mayo Clinic, USA

**Aims:-** Endostatin is a potent endogenous peptide that has a negative regulatory effect on endothelial cells (EC) but the mechanism remains unclear. This study was designed to assess the apoptotic and anti-proliferative effect of endostatin in stimulated and quiescent endothelial cells. **METHODS:-**Human umbilical vein endothelial cells (EC), were treated VEGF, and bFGF, and endostatin with and without VEGF and bFGF. After 24 hours incubation, apoptosis was measured by detecting intracellular nucleosomes and proliferation was quantified by the bromodeoxyuridine incorporation assay. **RESULTS:-**Endostatin alone increased apoptosis by 42 % and in the presence of VEGF and bFGF the apoptosis was reduced to 3 % and 15 % respectively *(p=0.02).* In EC treated with VEGF and bFGF, apoptosis was reduced by 25 % and 27 % respectively *(p=0.03).* Endostatin decreased the proliferative effect of VEGF, (36 % to 22 %) and bFGF (23 % to 8%). In unstimulated EC, endostatin alone had no effect on proliferation. **CONCLUSION:-**In quiescent EC, the anti-angiogenic effect of endostatin is mediated by inducing apoptosis. However in stimulated EC, endostatin has an anti-proliferative effect.

**P211**

**BLOCKADE OF VASCULAR ENDOTHELIAL GROWTH FACTOR RECEPTORS INCREASES TUMOUR CELL APOPTOSIS**

Martin P. Barr*, David J. Bouchier-Hayes, Judith H. Harmey.
Department of Surgery, Royal College of Surgeons in Ireland, Education & Research Centre, Beaumont Hospital, Dublin 9, Ireland.

Angiogenesis is critical for tumour growth and metastasis. Several angiogenic factors have been identified, including the particularly potent cytokine, VEGF (vascular endothelial growth factor). We have previously shown that VEGF acts as a survival factor for murine and human mammary adenocarcinoma cells by upregulating Bcl-2, thereby inhibiting apoptosis [1,2]. Antibodies raised against VEGF have been shown to suppress tumour growth in vivo[3], indicating that VEGF antagonists may have therapeutic applications as inhibitors of tumour-induced angiogenesis. To date, a number of VEGF receptors have been identified, namely Flt-1, Flk-1/KDR and more recently Neuropilin-1. In this study, we aimed to identify peptides blocking the binding of VEGF to its specific receptors on breast tumour cells.

Total RNA was isolated from murine (4T1) and human (MDA-MB-231) mammary adenocarcinoma cells and cDNA synthesised by reverse transcriptase for PCR using primers specific for the VEGF receptors Flt-1 and Flk-1/KDR. Human umbilical vein endothelial cells were used as a positive control. Neuropilin-1 expression was analysed by Western blot. Peptides were designed to block KDR and Neuropilin-1 receptors. Cells ($1x10^4$) were seeded in 96-well plates and treated with a repertoire of peptides for 24hr in the presence/absence of recombinant VEGF (10ng/ml). Tumour cell apoptosis was measured using the TUNEL assay (TdT-mediated dUTP nick end labelling).

Flt-1 and KDR receptors were expressed on human MDA-MB-231 tumour cells and Flt-1 in murine 4T1 tumour cells. A 130kD protein corresponding to Neuropilin-1 was identified in both tumour cell lines. Peptides directed specifically against the VEGF receptors Flk-1/KDR and Neuropilin-1 induced tumour cell apoptosis, an effect that was blocked by the addition of recombinant VEGF.

Our results identify an additional role for VEGF in tumour growth. In VEGF receptor-expressing tumour cells, anti-KDR and anti-Neuropilin-1 peptides increase apoptosis by blocking VEGF binding to its cognate receptors.

1 Pidgeon, GP., Barr, MP, Harmey, JH., Foley, DA., Bouchier-Hayes, DJ., 2001. Brit. J. Cancer, 85, 273.
2 Harmey, JH., Bouchier-Hayes, DJ., 2002. BioEssays (In Press).
3 Kim, KJ., Li, B., Winer, J., Armanini, M., Gillet, N., Philips, HS., Ferrara, N., 1993. Nature, 362, 841.

**P212**

**COX-2 INHIBITORS REDUCE VEGF PRODUCTION IN NORMOXIA AND HYPOXIA.**

G Roche-Nagle*, J Harmey, D Bouchier-Hayes. Department of Surgery, Beaumont Hospital, Dublin 9.

Tumour strategies must be effective in normoxic and hypoxic conditions. Vascular endothelial growth factor (VEGF) has been shown to be a survival factor for tumour cells and is up regulated by hypoxia. Cyclooxygenase-2 (COX-2) inhibitors decrease experimental breast cancer growth through a VEGF mediated pathway. We examined the effect of COX-2 inhibition on tumour cell expression of VEGF in a normoxia and hypoxia environment.

Murine (4T1) metastatic mammary carcinoma cells were treated with COX-1 inhibitor (SC-560), COX-2 inhibitor (NS-398) or indomethacin under normoxic and hypoxic conditions. VEGF was assayed by ELISA and expressed as pg /μg cell protein.

| Treatments | Normoxia | | Hypoxia | |
|---|---|---|---|---|
| Controls | 1.40 | | 2.58 | |
| Concentrations | *1μM* | *5μM* | *1μM* | *5μM* |
| Cox 1 inhibitor | 1.35 | 1.28 | 1.87 | 1.53 |
| Cox 2 inhibitor | 0.54*# | 0.75*# | 0.87*# | 0.81*# |
| Indomethacin | 1.08 | 0.68* | 1.24 | 0.79* |

*ANOVA; non parametric analysed with Kruskal Wallis; *=p<0.05 Mann-Whitney Test; # =p<0.05 N=9*

In 4T1 cells, hypoxia increased VEGF production 2-fold relative to controls. Indomethacin or the selective COX-2 inhibitor (NS-398) reduced VEGF production under normoxic and hypoxic conditions. They were found to be more sensitive to COX-2 under hypoxic conditions. COX-1 inhibition (SC-560) did not alter VEGF production. Thus COX-2 inhibition down regulates VEGF release from cancer cells under normoxic and hypoxic conditions, and as such satisfies an important requirement for an anti-cancer treatment.

**P213**

**PROTEOLYSIS OF HUMAN FIBULIN-1 IN BREAST CARCINOMAS AND *IN VITRO***

Lisa M. Greene[a*], Waleed O. Twal[b], Enda Mc Dermott[c], Niall O' Higgins[c], Michael J. Duffy[a], W. Scott Argraves[b], and William M. Gallagher[a,+]
[a]Conway Institute of Biomolecular and Biomedical Research, Department of Pharmacology, University College Dublin, Belfield, Dublin 4, Ireland. [b]Department of Cell Biology and Anatomy, Medical University of South Carolina, 173 Ashley Avenue, Charleston, South Carolina, USA. [c]Conway Institute of Biomolecular and Biomedical Research, Department of Surgery, University College Dublin, Belfield, Dublin 4, Ireland.

Fibulin-1 is a secreted glycoprotein and has been implicated in the regulation of tumour development. For example, fibulin-1 can suppress cell adhesion, motility and invasion of a variety of tumour cell lines *in vitro*, as well as

 © 2002 Cancer Research UK

extend the latency of fibrosarcoma tumour growth in nude mice. Fibulin-1 is an essential component of extracellular matrix (ECM) structures and may be subjected to proteolysis during tumour development. In this study, we examined the sensitivity of immunoaffinity-purified human fibulin-1 protein to proteolysis by MMP-3, –7 and other tissue proteases. Proteolysis of fibulin-1 was analysed by Western blotting using an N-terminal-specific monoclonal antibody. Fibulin-1 (100kDa) was readily cleaved by MMP-7 and thrombin in a dose- and time-dependant manner. Cleavage of fibulin-1 by thrombin produced a 50 kDa N-terminal fragment. A 30 kDa N-terminal fragment and a corresponding C-terminal 70 kDa fragment of fibulin-1 were detected following digestion with MMP-7. Digestion of fibulin-1 by plasmin and leucocyte elastase gave rise to a more extensive cleavage pattern resulting in fragments ranging from 70 - 30 kDa. Western blot analysis using the N-terminal-specific antibody was used to determine if proteolysis of fibulin-1 occurs *in vivo* in breast cancer. In addition to full-length fibulin-1, we identified several immunoreactive N-terminal fragments (55, 50, 30, and 25 kDa) in human breast tissue. The 55 kDa proteolytic fragment of fibulin-1 was detected in both normal and neoplastic breast tissue. However, the 50 kDa fragment of fibulin-1 was found more frequently in benign breast tumors (5/5, 100%) (Chi-square = 4.55, $P = 0.03$) and in breast carcinomas (35/39, 90%) (Chi-square = 17.22, $P < 0.0001$) than in normal breast tissue (6/18, 33%). Occurrence of the 25 kDa fragment was limited to just 5% of the breast carcinomas analysed. Immunoreactive fragments of fibulin-1 were also detected in the cell extract of 6 breast cancer-derived cell lines. These differences in terms of fibulin-1 proteolysis between normal and neoplastic breast tissue presumably reflect deregulated proteinase activity in the carcinomas. We have shown for the first time that human fibulin-1, a candidate tumour suppressor gene, is readily cleaved by a number of proteases *in vitro* and is proteolytically modified during breast cancer development. [Supported by the Irish Cancer Society, Enterprise Ireland and the Association for International Cancer Research (WG) and NIH Grant HL52813 (WSA)].

**P214**

**EFFECT OF CELL CYCLE ON CELLULAR HIF-1α LOCALISATION**

Gabi U. Dachs*, Chryso Kanthou, Olga Greco, Claudia Coralli and Gillian M. Tozer
Gray Cancer Institute, Mount Vernon Hospital, Northwood, HA6 2JR

The field of hypoxic gene regulation has expanded exponentially in recent years. An oxygen responsive enhancer sequence (hypoxia-responsive element, HRE) and its corresponding transcription factor (hypoxia inducible factor 1, HIF-1) have been identified (reviewed in Dachs and Tozer, 2000, Eur J Cancer 36, 1649). HIF-1 is a heterodimer consisting of the subunits HIF-1α and HIF-1β, both belonging to the basic-helix-loop-helix per-aryl hydrocarbon receptor nuclear translocator-sim family of transcription factors. HIF-1 is common to all mammalian cells tested to date and has also been detected in all human tissue and organs assayed. Several recent studies demonstrated that the majority of clinical tumour specimens overexpressed the HIF-1α protein subunit. Controlling gene expression via the HIF-1/HRE system has therefore been proposed as a way of targeting gene therapy to solid tumours (Dachs *et al*., 1997, Nature Medicine 3, 515).

The activation of HIF-1α is a multistep process of hypoxia-dependent nuclear import, de-repression of the activation domain and recruitment of the p300 coactivator. The amount of HIF-1α protein increases during hypoxia and is degraded rapidly during reoxygenation in intact cells. HIF-1α and HIF-1β bind in the cytosol and dimerisation is required for stable association with the nuclear compartment. We have analysed the cellular localisation of HIF-1α by immunofluorescence in T24 bladder carcinoma, FaDu nasopharyngeal squamous carcinoma and MDA 231 breast carcinoma cells. A variation in staining and nuclear localisation was observed. Specifically, cells undergoing mitosis lost nuclear localisation following induction by the hypoxia mimetic agent, $CoCl_2$. Distribution of HIF-1α throughout the cell cycle was analysed using FACS. The effect of overexpression of HIF-1α, HIF-1β, EPAS, p300 and CBP on HIF-1α localisation following transient transfection of T24 cells were also analysed.

Reporter gene analysis has shown that synthetic promoters containing HRE sequences derived from PGK1 (5x 18 bp) and VEGF (5x 18 bp) responded not only to hypoxic stimuli, but also to radiation (5 Gy) in transiently transfected T24 cells (Greco *et al*, submitted manuscript). The effect of ionising radiation on HIF-1α localisation was therefore determined.

This work was funded by Cancer Research UK.

**P215**

**DYNAMIC CONTRAST ENHANCED MRI ASSESSMENT OF ACUTE MICROVASCULAR EFFECTS WITH CONVENTIONAL CYTOTOXIC CHEMOTHERAPY IN HUMAN TUMOURS.**

*KJ Lankester[1], NJ Taylor[2], JJ Stirling[2], J Boxall[1], GJS Rustin[1], MO Leach[3], RJ Maxwell[4], AR Padhani[2]. [1]Dept of Medical Oncology, Mt Vernon Hospital, Northwood Middx, HA6 2RN. [2]Paul Strickland Scanner Centre, Mt Vernon Hospital. [3]MRI Unit, Royal Marsden Hospital, Sutton, SM2 5PT. [4]Gray Cancer Institute, Mt Vernon Hospital, Middx, HA6 2JL.

**Background:** Vascular targeting agents, such as combretastatin, cause acute reductions in tumour blood flow resulting in necrosis in animal models[1]. Dynamic contrast enhanced MRI (DCE-MRI) kinetic parameters related to tissue permeability and perfusion can be used to quantify these effects ($K^{trans}$: volume transfer constant between blood plasma and the extravascular extracellular space (EES) and $v_e$: volume of EES per unit volume of tissue). In a recent phase I trial, significant changes in $K^{trans}$ and $v_e$ were seen 24 hours after combretastatin using DCE-MRI[2]. Combretastatin will be used in combination with cytotoxic agents in future trials. The latter are not thought to have specific acute effects on tumour vasculature. However, this has not been verified, and it will be important to distinguish the effects of combretastatin from any unexpected vascular contributions from the cytotoxic agent. Thus, the purpose of this study was to observe if cytotoxic agents have any acute effects on the tumour vasculature using DCE-MRI. Taxane and platinum based regimens were chosen as they are the likely choices for future trials.

**Methods:** Three scans were done on consecutive days - 2 pre-treatment (for repeatability) and 1 post-treatment. Serial T1-weighted dynamic images were obtained before and after the bolus administration of IV Gd-DTPA (0.1 mmol/kg). Chemotherapy was given after the 2$^{nd}$ scan. The average time from the start of chemotherapy to the 3$^{rd}$ scan was 21 hours. Repeatability statistics derived from the measurements on days 1 & 2 for the group were used to assess the effect of chemotherapy on day 3 for individual patients. The following statistics were used: the mean squared difference for the group (dSD); within patient standard deviation (wSD); within patient coefficient of variation (wCV); repeatability (r) [3].

**Results:** 9 women with gynaecological tumours have been imaged so far and 7 were suitable for analysis. The average age was 56 years old and all had WHO performance status ≤2. The repeatability value for $\log_{10}K^{trans}$ was –33.6% to +50.7% and ±11.8% for $v_e$ (wSD for $\log_{10}K^{trans} = 0.0643$ and for $v_e = 0.0427$). So far, from the comparison of the day 3 measurement with the mean value of days 1 & 2, there has been no significant change in $K^{trans}$ for any patient and no significant change in $v_e$ for 5/7 patients within 24 hours of the first cycle of conventional chemotherapy.

**Conclusion:** Results to date show no systematic changes in kinetic parameters related to perfusion/permeability. Further data are being acquired and we aim to recruit a total of 20 patients.

**References:**

1. Chaplin DJ et al. Br J Cancer 1999;80(S1):57
2. Galbraith SM et al, Proc ASCO 2001:20:70a
3. Bland JM & Altman DG. BMJ 1996;313:106

**P216**

**TUMOUR XENOGRAFT REGRESSION CAN BE INDUCED BY COMBINING THE VASCULAR-TARGETING AGENT ZD6126 WITH THE VEGF RECEPTOR TYROSINE KINASE INHIBITOR ZD6474.**

A.J. Ryan*, J. Kendrew, D.J. Ogilvie, L.F. Hennequin, S.R. Brave, S.E. Ashton, J.A. Calvete, D.C. Blakey and S.R. Wedge. *Cancer and Infection* Research, AstraZeneca, Alderley Park, Macclesfield, Cheshire, SK10 4TG, United Kingdom.

© 2002 Cancer Research UK

Anti-angiogenic and vascular targeting approaches are focussed upon exploiting differences between the endothelium of tumours and normal tissues to achieve a therapeutic anti-tumour effect. However, these approaches are mechanistically distinct: anti-angiogenics are intended to limit tumour growth and progression by preventing recruitment of new tumour blood vessels, while vascular-targeting agents aim to disrupt pre-existing tumour vasculature, leading to vessel occlusion and the induction of extensive tumor necrosis. Given their different mode of action, a combination of these two approaches may prove complementary.

Since VEGF has a pivotal role in inducing pathological angiogenesis, inhibition of VEGF-signalling is viewed as a key anti-angiogenic strategy. We have developed the orally-active VEGF receptor tyrosine kinase inhibitor ZD6474 (Wedge S.R. et al., Proc. Amer. Assoc. Cancer Res., 42: Abst. 3126, 2001) and shown it to elicit broad-spectrum anti-tumour activity in a range of models following chronic once-daily administration. The current study examined ZD6474 in combination with the novel vascular targeting agent ZD6126, which causes selective disruption of tumor vasculature (Blakey, D.C. et al., Proc, Amer. Assoc. Cancer Res., 42: Abst. 4421, 2001). ZD6474 (50 mg/kg/day, p.o.) or vehicle was administered to mice bearing established Calu-6 human lung tumors (0.7 $cm^3$ volume) for two weeks, with or without a single dose of ZD6126 (100 mg/kg, i.p.) given on day 7. ZD6474 alone inhibited tumor growth significantly (73%, p = 0.001 by one-tailed t-test) while ZD6126 alone produced a transient growth delay (6% inhibition at the end of the experiment, p = 0.4). However, a combination of ZD6474 and ZD6126 inhibited growth of tumors completely (> 100%) with evidence of tumor regression post-ZD6126 administration. Similar experiments were performed in established Lovo human colorectal tumors (0.5 $cm^3$ volume) with daily administration of ZD6474 (50 mg/kg/day, p.o.) for 11 days and acute administration of ZD6126 (100 mg/kg, i.p., day 5). The combination again produced a greater effect than either agent alone, inhibiting tumor growth completely and inducing tumor regressions. These preclinical experiments do suggest that a combination of anti-angiogenic and vascular-targeting agents may provide added therapeutic benefit.

### P217
### CHARACTERISATION AND QUANTIFICATION OF AN ENDOTHELIAL CELL TUBE FORMATION ASSAY

S.R. Brave*[1], A. Bigley[2], J. Kendrew[1], S. Cornforth[1] & S.R. Wedge[1].
Departments of [1]Cancer and Infection Bioscience and [2]Safety Assessment, AstraZeneca, Alderley Park, Macclesfield, Cheshire, SK10 4TG, UK.

Angiogenesis, the formation of new blood vessels from pre-existing vasculature, plays a significant role in a number of pathological conditions, including tumour growth (Folkman & Shing, J. Biol. Chem, 267; 10931, 1992). This complex multi-step process involves endothelial cell migration, proliferation and differentiation into tubules. We have examined an *in vitro* model of endothelial cell tube formation (AngioKit, TCS Biologicals, UK) with the aim of characterising levels of 3 angiogenic stimuli (VEGF, bFGF and IL-8) during the course of the assay. HUVECs and human fibroblasts were obtained as co-cultures (day 1; AngioKit). Cells were maintained at $37^0C$ with 5% $CO_2$ and the medium changed every 2-3 days. Aspirated media was stored for analysis of VEGF, b-FGF and IL-8 using ELISA.

| pg/ml ± s.e.m | Basal medium + b-FGF | | Basal medium - b-FGF | |
|---|---|---|---|---|
| | Day 1 | Day 11 | Day 1 | Day 11 |
| VEGF | 85 ± 1.2 | 2000 ± 105 | 0 | 1300 ± 70 |
| bFGF | 400 ± 20 | 75 ± 18 | 0 | 0 |
| IL8 | 100 ± 14 | 1375 ± 72 | 115 ± 55 | 95 ± 15 |

In the presence of exogenous b-FGF (~700pg/ml), high levels (> 1 ng/ml) of endogenous VEGF and IL-8 were apparent in the incubation media harvested on day 11. Under these conditions vessel growth in response to additional exogenous VEGF (20ng/ml) was low. In comparison, the removal of exogenous b-FGF from incubation media resulted in a 93% reduction in IL-8 and a 35% reduction in VEGF (in media harvested at day 11). This enabled a greater response to exogenous VEGF to be demonstrated: conditions that were subsequently used to examine inhibitors of VEGF-receptor signalling.

To quantify tubule growth a novel whole-well image analysis methodology was developed using a Zeiss KS400 3.0 image analyser (Imaging Associates Ltd). Tubule formation was examined at day 11 following fixing and staining of tubules for CD31. The morphological parameters measured were total area of tubule growth, total tubule length, number of bifurcations, and total area of clusters (un-migrated cells). Small molecular weight inhibitors of VEGF-receptor tyrosine kinase activity have been investigated against each of these parameters.

### P218
### A NOVEL APPROACH TO ASSESSING RESPONSE TO CHEMOTHERAPY USING HYBRID CAMERA FDG PET

[1*]RJ Francis, [1]AJ Green, [1]S Baig, [1]V Knell, [2]I Frisken, [1]RL Bowen, [3]AJW Hilson, [3]JR Buscombe, [1]RHJ Begent
[1]Cancer Research UK Targeting and Imaging Group, Department Oncology, Royal Free and University College Medical School, London NW3 2PF
[2]Radiology Department and [3]Department of Medical Physics , Royal Free Hospital, London NW3 2QG

**Aim:** Response to chemotherapy in solid tumours is conventionally assessed by dimensional measurements of CT scans after 3 months of treatment. Studies have shown that 18-F fluorodeoxyglucose positron emission tomography (FDG PET) on a dedicated PET (DPET) system may be able to predict response earlier than CT scans, however there are limitations with the current methods of quantitative analysis. Response assessment has not yet been examined with hybrid camera PET (HCPET). HCPET is more affordable and can be used for routine nuclear medicine imaging, but has lower resolution than DPET. HCPET has the advantage that it could be widely available in both cancer centres and cancer units.

**Method:** 25 patients with metastatic disease having first or second line chemotherapy entered a study comparing FDG PET (HCPET) to CT scans and serum tumour markers for response assessment. FDG PET, CT scans and serum tumour markers were obtained pre-treatment and after 3 months of chemotherapy. An additional FDG PET scan was performed at 2-4 weeks after treatment to assess its predictive value. A novel method of semi-quantitative analysis of the FDG PET scans has been developed to automatically define tumour volumes (segmentation) in an objective, reproducible and reliable manner. These segmented images are then analysed using count density histograms, which provide both a numerical and pictorial display of functional tumour changes with therapy. CT scans were measured according to the RECIST criteria.

**Results:** All patients who attained a partial response on CT at 3 months had a 15% or greater reduction in tumour FDG uptake at the 2-4 week time-point. No patients with progressive disease on CT at 3 months showed a 15% or greater reduction in tumour FDG uptake. All patients with radiological stable disease who had a reduction in tumour FDG uptake at 2-4 weeks also had a fall in tumour markers. This may indicate a biological response to therapy that is not being detected by anatomical measures.

**Conclusion:** HCPET shows potential for predicting response to chemotherapy after only 2-4 weeks of treatment. The FDG PET scan results and the changes observed in serum tumour markers suggest that chemotherapy may be producing a biological change which is not being detected by dimensional measurements alone.

This study has approval from the Royal Free Hospital Local Research Ethics Committee (LREC) and the Administration of Radioactive Substances Advisory Committee (ARSAC).

### P219
### DIFFERENTIAL EXPRESSION OF CA-IX IN HUMAN CARCINOMA CELL LINES

CA Parker*[1], M Jaffar[1], KJ Williams[1], AL Harris[2], IJ Stratford[1].
[1]Experimental Oncology Group, School of Pharmacy and Pharmaceutical Sciences, University of Manchester, Oxford Road, Manchester, M13 9PL.
[2]Molecular Oncology Group, Institute of Molecular Medicine, John Radcliffe Hospital, Oxford, OX3 9DU.

CA-IX is a member of the α class of carbonic anhydrases (CA) that catalyse the hydration of $CO_2$ ($H_20 + CO_2 \leftrightarrow H^+ + HCO_3^-$) and serve a wide range of physiological functions including acid-base regulation and respiration. CA-IX is tumour-associated and may have potential roles in oncogenesis. CA-IX is upregulated by hypoxia, which is associated with increased aggressiveness,

 © 2002 Cancer Research UK

metastasis and poor prognosis. It has been postulated that CA-IX is involved in maintaining the extracellular acidification common to solid tumours that is assumed to be related to enhanced proliferation, invasiveness and has implications for chemotherapeutic drug uptake and activity. The aims of the study were to assess the effect of anoxia for inducing CA-IX protein expression in a panel of cell lines, to identify if expression of CA-IX is related to cell growth and finally determine whether CA-IX expression can reflect itself in changes in drug uptake and toxicity.
A panel of 16 cell lines was characterised for CA-IX protein expression under both aerobic and anoxic conditions by western blotting. The growth of MDA435 cells transfected with *CA-9* and empty vector controls was assessed under aerobic and anoxic conditions by cell counting.
The levels of CA-IX varied between cell lines from HT1080 and RT112 cells expressing very low levels to high expression in SW480 cells in aerobic conditions. A subset of the panel exhibited substantial anoxic induction of CA-IX (e.g. H647, HT29, MDA231, SW480). CA-IX expressing MDA435 cells showed reduced growth compared to controls in air, however the inhibition was absent in anoxia.
The majority of tumour cell lines tested expressed CA-IX to varying degrees but not all exhibited induction by anoxia, suggesting that the effect may be cell type specific. Expression of CA-IX did not appear to provide a growth advantage to tumour cells under aerobic or anoxic conditions. Further studies will be carried out in which a selected panel of cells with high and low CA-IX expression will be treated with a known CA inhibitor (acetazolamide) in combination with anticancer agents.

**P220**

**ALTERED SENSITIVITY TO HYPOXIA IN PROSTATE CANCER CELL LINES; HYPOXIA-INDUCED APOPTOSIS A MITOCHONDRIAL INDEPENDENT EVENT.**

Ronan N.T. Coffey*, R. William G. Watson, Cormac T. Taylor, John M. Fitzpatrick
Department Surgery, Mater Misericordiae Hospital, Conway Institute, University College Dublin, Ireland.

Hormone manipulation (androgen ablation) is the most effective treatment for advanced prostate cancer to date. This treatment however is palliative, as only a proportion of prostate cancers cells are dependent upon androgen for survival. Following androgen ablation, patients eventually relapse to an androgen independent state. At the molecular level, many factors have been implicated with the emergence of androgen independent prostate cancer. The ability of prostate cancer cells to survive in a hypoxic environment may be one of these contributing factors. The objectives of this study therefore were to assess the effects of hypoxia on apoptosis in an androgen sensitive LNCaP and insensitive PC-3 prostate cancer cell line; to identify a role for the caspases, a family of cell death proteases in this process.
Cells were grown under hypoxia (1-5% $0_2$ , 0- 72 hrs). Apoptosis was assessed by sub $G_0$ propidium iodide incorporation and Annexin V staining. Hypoxic inducible factor-1 alpha (HIF-1α) expression and mitochondrial cytochrome c release to the cytosol were assessed by Western blotting. Caspase activity was assessed using caspase 3, 6/8 and 9 AMC-tagged fluorescent substrates.
The LNCaP cell line displayed no basal expression of HIF-1α under normoxia, but did undergo induced expression at 24-48 hrs hypoxia. The PC-3 cell line on the other hand, constitutively expressed HIF-1α under both normoxia and hypoxia. Hypoxia resulted in cell death in the LNCaP cells with maximum percent apoptosis occurring at 72 hrs 1% hypoxia (28.4±2.1%) compared to normoxia (10.5±1.7%). PC-3 cells remained viable at 72 hr 1% hypoxia and displayed no increase in percent apoptosis (6.1±0.6% normoxia compared to 5.2±0/6% 72 hr 1% hypoxia). Apoptosis in the LNCaP cell line was associated with caspase 3 and 8 activation but was independent of caspase 9. This was confirmed by the inability to detect the release of cytochrome c from the mitochondria to the cytosol in the LNCaP cell line, a rate-determining step in the activation of caspase 9.
We demonstrate that hypoxia elicits an apoptotic response only in the androgen sensitive LNCaP prostate cancer cell line, in what appears to be a mitochondrial and caspase 9 independent manner. The ability of prostate cancer cells to grow in a hypoxic environment may a contributing factor leading to the development of the hormone refractory state. The constitutive expression of HIF-1α may represent an important survival factor in PC-3 cells which are inherently more resistant to hypoxia-induced apoptosis than the androgen sensitive LNCaP cell line. The role HIF-1α plays in the survival signaling of both cell lines remains to be fully elucidated and is currently under investigation.

**P221**

**UP-REGULATION OF THE p75 DEATH RECEPTOR FOLLOWING EXPOSURE OF ESFT CELLS TO bFGF.**

Jim Boyne*, Ben Dibling and Susan Burchill.
Candlelighter's Children's Cancer Research Laboratory, Cancer Research UK Clinical Centre, St James's University Hospital, Leeds, LS9 7TF, UK.

Our previous experiments have shown that treatment of tumours of the Ewing's sarcoma family (ESFTs) with basic fibroblast growth factor (bFGF)-induces cell death (Sturla et al, 2000, Cancer Research, 60(1), 6160-6170). bFGF-induced cell death is associated with activation of the initiator caspases -2, -8 and –10 (Westwood et al, 2002, Oncogene, 21(5):809-824), which are most frequently cleaved by members of the TNF super-family of receptors. In this study we have investigated the hypothesis that bFGF-induced cell death may be mediated through the cell death receptor p75.
The effect of bFGF (20ng/ml) has been examined in 6 ESFT cell lines. Phosphorylation of FGFR1 and activation of the downstream signalling molecules Ras and ERK was investigated by immunoprecipitation, GST binding assay and Western blot respectively. The effect of bFGF on p75 expression was also determined by Western blot.
bFGF induced cell death in 4/6 cell lines studied and was independent of *EWS-ETS* gene rearrangement type. Phosphorylation of FGFR1 was observed within a 5 min exposure of ESFT cells to bFGF. This was accompanied by phosphorylation of a co-precipitated protein (90kD), consistent with that of the FGF docking protein FRS2, and activation of the downstream signalling molecules Ras and ERK. Up-regulation of the death receptor p75 occurred 24h after exposure to bFGF in ESFT cells, this preceded cleavage of caspases –2, -8 and –10 (48h) and cell death (48-72h) (Westwood et al, as above). However expression of p75 was unchanged in ESFT cells that do not die when exposed to bFGF.
In summary, bFGF-induced cell death appears to be initiated by phosphorylation of FGFR1 and rapid activation of the downstream signalling proteins Ras and ERK. This precedes up-regulation of the death receptor p75, which may be transcriptionally regulated by bFGF. These data are consistent with an ordered cell death pathway, which could offer novel targets in the development of therapeutic treatments for ESFTs.

**P222**

**ANGIOGENESIS IN ANAL WARTS, ANAL INTRAEPITHELIAL NEOPLASIA (AIN) AND ANAL SQUAMOUS CELL CARCINOMA**

*J. Mullerat[1], L.F. Wong Te Fong[2], S. E. Davies[3], M.C. Winslet[1], C.W. Perrett[2]
Departments of [1]Surgery, [2]Obstetrics and Gynaecology, [3]Histopathology, Royal Free campus, Royal Free and University College Medical School, London, U.K.

**Introduction**:
Squamous cell carcinoma (SCC) of the anus is a rare malignancy, but it is becoming increasingly more common. Most cases of anal carcinoma seem to develop from dysplastic anal lesions (anal intraepithelial neoplasia - AIN) caused by persistent anal warts. In this study we examined biopsies of anal lesions ranging from warts to invasive anal carcinoma, with the aim to compare the degree of angiogenesis between anal warts, AIN and anal SCC.
**Method**:
Samples from 70 patients (56 males and 18 females) who had undergone excision biopsy or resection of anal warts (20), AIN I (12), AIN II-III (25) and anal SCC (13) were studied. The samples were stained for von Willebrand factor (vWF), a specific marker of endothelial cells. Angiogenesis was measured by microvessel density (MVD), quantifying the microvessels in the stroma adjacent to the epithelial lesion, by 3 different observers.
**Results:**
Average microvessel counts and standard deviation values were as follows: 6.30 ± 2.50 for warts; 8.34 ± 2.83 for low-grade AIN; 16.28 ± 3.92 for high-

© 2002 Cancer Research UK

grade AIN and 23.85 ± 6.01 for SCC. These results show a statistically significant increase ($p<0.001$) in MVD in high grade anal intraepithelial neoplasia (AIN II-III) compared to low grade anal intraepithelial neoplasia (AIN I) and in anal SCC compared to AIN II-III.

**Conclusion:**

There is increased angiogenesis in in high grade AIN, compared to low grade AIN and anal warts. These findings suggest that there are progressive abnormal patterns of microvascularisation at early stages of anal carcinogenesis.

**P223**

**HOW ACCURATELY CAN THE ONCOLOGIST REPORT FDG-PET TUMOUR RESPONSE USING THE ADAC VERTEX PLUS DUAL-HEADED GAMMA CAMERA?**

RL Bowen*[1], RJ Francis[1], AJ Green[1], S Baig[1], V Knell[1], JR Buscombe[2], AJW Hilson[2], RHJ Begent[1]

[1]CRC Targeting and Imaging Group, Department of Oncology, Royal Free Campus, Royal Free & University College Medical School, UCL, London, NW3 2PF

[2]Department of Nuclear Medicine, Royal Free Hospital, London, NW3 2PF

**Purpose**: We compared the visual reports of two experienced Nuclear Medicine Consultants and those of an oncology research fellow with no previous FDG-PET experience and minimal training, to assess whether this method of detecting tumour response can be reliably reported by the oncologist.

**Methods**: Twenty-two patients with unresectable, locally recurrent or metastatic colorectal, oesophageal, hepatocellular, pancreatic and breast carcinoma receiving conventional chemotherapy were assessed with FDG-PET scans at 0, 2-4 and 8-12 weeks. FDG-PET scans were performed on an ADAC Vertex Plus Dual Headed Gamma Camera. Twenty one pairs of scans were assigned random numbers and temporal order and the three investigators were required to report stable disease, response or progressive disease while blinded to the patient details, the natural order of the scans and the CT and tumour marker responses. The actual tumour response at each time point was determined by combining FDG-PET appearances, CT scan and tumour marker responses and patient follow-up.

**Results**: Both experts reported the scans accurately in 76% and the novice reported the correct FDG-PET response in 81% of cases. The nuclear medicine physicians concurred with each other in 76% of cases although they were not always accurate in the same scan pairs. The novice concurred with both nuclear medicine physicians in 62% (13/21) of cases. Of these 13 scan pairs the accurate report differed in only one. In one partial response all three investigators reported stable disease. Where there was no concordance with the experts the novice was correct in 63%(5/8). At least one of the experts reported accurately in 100% (3/3) of the scan pairs where the novice was incorrect.

**Conclusion**: Our results confirm that FDG-PET is a robust technique for assessing tumour response to chemotherapy. This could have wide reaching benefits for cancer centres and units where the more affordable Dual-Headed Gamma Camera could be used routinely. Report reliability may be further improved with the use of a method for quantitatively assessing tumour uptake of FDG and therefore response.

**P224**

**PHAGE DERIVED-ANTI-VEGF-RECEPTOR-2 (VEGF-$R_2$) ANTIBODY IN COMBINATION WITH METRONOMIC ESTRAMUSTINE : A NOVEL STRATEGY FOR INHIBITING ANGIOGENESIS**

T. Lam*[1], J. Greenman[1], C. Newton[1], J. Hetherington[2] & A. Maraveyas[2]

[1]Medical Research Laboratory, University of Hull, Hull HU6 7RX;

[2]Academic Department of Uro-oncology, Princess Royal Hospital, Hull HU8 9HE.

VEGF-receptor-2 has been proposed as a rational target of anti-angiogenic therapy, due to its fundamental role in mediating the mitogenic effects of VEGF on vascular endothelial cells. Metronomic dosing is a new approach in the treatment of cancer whereby low concentrations of cytotoxic drugs with strong anti-angiogenic actions are given continuously in combination with an anti-angiogenic biological agent to optimize inhibition of angiogenesis whilst minimizing systemic toxicity. However, the ideal agents for such a therapeutic strategy remain unclear.

We are in the process of developing a novel anti-angiogenic treatment strategy combining an anti-VEGF-$R_2$ blocking antibody derived from phage display technology with metronomic dosing of estramustine, an orally bioavailable cytotoxic agent with anti-mitotic actions.

Single-chain antibodies specific for VEGF-$R_2$ were isolated using a phage display library after 5 rounds of biopanning against recombinant VEGF-$R_2$/Fc chimera immobilized on immunotubes, and against the same antigen suspended in solution coupled to magnetic beads. Further selection is being performed using ELISA, with VEGF-$R_1$ as negative control, and through competitive binding with VEGF, hence selecting a VEGF-$R_2$ specific antibody capable of blocking the binding of VEGF to VEGF-$R_2$. Endothelial cytotoxicity of the blocking antibody and estramustine is being assessed using a human umbilical vein endothelial cell culture model, with growth (cell count and DNA replication), biological (formation of vascular networks and changes in VEGF-$R_2$ activation) and survival (DNA fragmentation ELISA) parameters being determined as end-points.

The combination of metronomic dosing of estramustine with an anti-VEGF-$R_2$ blocking antibody represents a new and potentially powerful anti-angiogenic treatment strategy. Benefits include high efficacy of anti-angiogenic effect due to the postulated synergy between the two agents based on enhancing endothelial cell apoptosis, high specificity due to the targeted action of the blocking antibody, ease of administration of estramustine (oral) and possibly low systemic toxicity due to the use of low doses of both agents. This approach is likely to have future implications for the treatment of traditionally chemo-resistant and highly vascular tumours such as advanced renal cell carcinoma.

**P225**

**COMPARISON OF THE THERAPEUTIC RESPONSE IN LEUKAEMIC GUINEA PIGS BETWEEN MODULATING AND NON-MODULATING ANTIBODY DERIVATIVES**

A.M. Smith[1], K.S. Kan[2*] & G.T. Stevenson[1], [1]Tenovus Research Laboratory, Southampton University Hospital, Tremona Road, Southampton SO16 6YD.

[2]School of Biological & Applied Sciences, University of North London , Holloway Road, London N7 8DB

A murine monoclonal antibody, 5G10, has been raised against the strain 2 guinea pig lymphocytes in the Institute of Tenovus. Using indirect flow cytofluorimetric analysis, 5G10 IgG antibody was found to remain on the surface of target cells over a two-hour incubation period. On the contrary, the % of initial fluorescence of a modulating monoclonal antibody, RJD IgG, was found to decline dramatically over the same incubation period. This shows that bound 5G10 IgG antibody is not internalised by the target cells.

The therapeutic response in leukaemic strain 2 guinea pigs was compared between the 5G10 non-modulating antibody derivatives and RJD modulating antibody derivatives. In this study, mouse/human chimeric antibody derivatives were synthesised in which the Fc is of human origin. Two types of antibody constructs, namely univalent $FabFc_2$ and bivalent $Fab_2Fc_2$, were produced from both 5G10 and RJD. In the therapy, groups of 10 age-matched guinea pigs were inoculated i.p. with 5 x $10^4$ $L_2C$ leukaemic cells. 24 hours after the inoculation, 4 mg of one of the four antibody derivatives was injected i.v. into each animal and their survival over 30 days was recorded. 5G10 bivalent $Fab_2Fc_2$ showed a significant therapeutic response whereas 5G10 univalent $FabFc_2$ produced no therapeutic effects. On the contrary, RJD univalent $FabFc_2$ showed much better therapeutic results over the RJD bivalent $Fab_2Fc_2$. These observations show that therapeutically, bivalent antibody constructs derived from non-modulating antibodies outperform the equivalent univalent constructs. On the contrary, univalent constructs derived from modulating-antibodies outperform the corresponding bivalent derivatives. Therefore, when designing antibody constructs for cancer immunotherapy, these are the important factors to consider.

© 2002 Cancer Research UK

**P226**

**PROFILING PROTEINS IN CELLS AFTER TREATMENT WITH 5-AZA-2 DEOXYCYTIDINE (DECITABINE)**

Debra Stuart*, Chris Twelves, and Robert Brown.
Department of Medical Oncology, Alexander Stone Building, Garscube Estate, Switchback Road, Bearsden, Glasgow University, Glasgow, UK

The DNA methyl transferase inhibitor 5-aza-2-deoxycytidine (Decitabine) can sensitise ovarian and colon tumour cell xenografts to a number of chemotherapeutic agents by demethylating DNA and reversing gene promoter methylation (Plumb, *et al* Cancer Res 60:21 6039-6044, 2000). This sensitivity correlates with re-expression of the mismatch repair protein hMLH1. Treatment of the cisplatin resistant human ovarian cell line A2780/cp70 for 24hrs with 1μM Decitabine induces the expression of a number of previously unexpressed gene products, including hMLH1, after 7 days in culture. Using two dimensional electrophoresis we have identified 31 distinct protein spots that are consistently present in cells exposed to Decitabine, but absent in control treated cells. In comparison, we have identified 15 protein spots present in control cells but lost from cells after treatment with Decitabine. Matrix Assisted Laser Desorption Mass Specrometry (MALDI-MS) and immunoblotting are being used to further characterise the protein changes induced by Decitabine in A2780/cp70.
Decitabine is currently being evaluated as an anti-cancer agent in clinical trials, both alone, and in combination with other therapeutic agents. Changes in protein expression have been analysed in peripheral blood mononuclear cells and skin samples from mice pre and post treatment with Decitabine. Results suggest analysis of these tissues may be an effective way of monitoring protein changes after exposure of cells in vivo to Decitabine.

**P227**

**TELOMERASE-DIRECTED GENE THERAPY OF HUMAN CANCER CELLS USING THE BACTERIAL NITROREDUCTASE GENE.**

Alan Bilsland[1]*, Jane A. Plumb[1], Rania Kakani[1], Jiangquin Zhao[1], Rosalind M. Glasspool[1], Richard J. Knox[2], T. R. Jeffry Evans[1] and W. Nicol Keith[1].
[1] Department of Medical Oncology, University of Glasgow, Cancer Research UK Beatson Laboratories, Garscube Estate, Switchback Road, Bearsden, Glasgow G61 1BD.
[2] Enact Pharma Plc, Porton Down Science Park, Salisbury SP4 OJQ.

Re-activation of human telomerase activity maintains telomere function, compensating for cell-division associated telomere attrition and is considered an essential late stage in immortalisation of human tumour cells. Since the association of human telomerase activity with cancer cells is highly specific and the RNA (hTERC) and protein (hTERT) components of telomerase are differentially regulated on a transcriptional level between normal and cancer cells, telomerase promoters may be useful in targeted gene therapy for the treatment of human malignancy. We used hTERC and hTERT promoter luciferase-reporters to quantify promoter activities in a panel of normal, cancer and ALT cell lines derived from lung, ovary, cervix, bladder and colon. Large differences were observed in promoter activities between normal and cancer cells, with all cancer cell lines tested showing higher promoter activity than the mortal cell strains tested. These differences were in the range of 40-300-fold for the hTERC promoter, while the hTERT promoter had a high activity in several cancer cell lines (29-833 light units) but did not appear above background in the mortal cell strains. We therefore selected a number of high and low expressing cell lines for inclusion in a stable cell line model of telomerase directed suicide gene therapy using the bacterial nitroreductase (NTR) gene which converts the pro-drug CB1954 to a cytotoxic form. We used MTT assay to quantify the IC50 value for the cytotoxic effect of CB1954 on cell lines harbouring hTERC-NTR, hTERT-NTR, CMV-NTR and promoter-less NTR constructs. The telomerase promoters could drive sufficient NTR expression to sensitise five human cancer cell lines with high promoter activity to the effects of bio-activated CB1954. In sensitive cell lines, the hTERC-NTR construct resulted in a range of sensitisation between 6-fold (lung adenocarcinoma cells) and 19-fold (ovarian adenocarcinoma cells) and the hTERT-NTR construct resulted in fold-sensitisation values between 5-fold (ovarian adenocarcinoma) and 14-fold (colorectal adenocarcinoma cells). The effect was dependent on high promoter activity and correlated with northern blots for NTR. We next addressed whether the vectors could sensitise cancer cells to CB1954 *in vivo*. Xenograft models of telomerase-NTR, CMV-NTR and negative control stable cell line sets of small cell lung cancer and cervical carcinoma cells were established and mice bearing tumours were given a single tail-vein injection of 40 or 80mg/kg CB1954. Tumour volumes were monitored for 7 days. hTERC- and hTERT-NTR tumours showed 63% and 76% reduction in the cervical model and 90% and 97% reduction in the lung model. Thus, telomerase directed gene therapy is an attractive approach for further development.

**P228**

**DESIGN, SYNTHESIS AND EVALUATION OF A STRUCTURALLY NEW CLASS OF TOPOISOMERASE I INHIBITORS: OXA-AZA-BENZO[*de*]ANTHRACENES**

A Di Salvo*, G Barsacq, D J Mincher, G Kay, Anticancer Drug Design and Delivery Research Group, School of Life Sciences, Napier University, Edinburgh, EH10 5DT, UK.

Human DNA topoisomerase I has been shown to be a valid target for anticancer therapy and inhibitors of this enzyme include the clinically active camptothecin derivatives topotecan and irinotecan. The clinical effectiveness of the camptothecin class of compounds is limited by the rapid *in vivo* conversion to inactive metabolites as a result of inherently labile structural features of the active drug molecules[1].
Towards the design of non-camptothecin inhibitors of topo I with increased structural stability we report the rational design and synthesis of a series of oxa-aza-benzo[*de*]anthracenes with angular ring systems that possess limited ability to intercalate into DNA and have enhanced DNA groove binding capability: features that favour interaction with topoisomerase I.

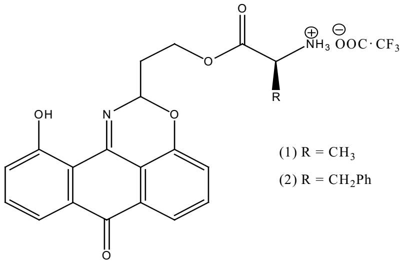

The 2*H*-3-oxa-1-aza-benzo[*de*]anthracen-7-ones **(1)** and **(2)** are representatives of a new class of topo I inhibitor with cytotoxic activity against human and animal cell lines *in vitro*; for example the L-alanine conjugate **(1)** is active against the human leukaemic HL60 cell line ($IC_{50}$ 7μM) and completely inhibited the topo I-mediated relaxation of supercoiled pBR322 DNA at 50μM as shown by changes in the electrophoretic mobility of the plasmid *in vitro*.
The chemosensitivity and enzyme inhibitory properties are modulated by the nature of the amino acid side-chain (R-group). Correlations are drawn between chemical structure, cytotoxic potency, DNA binding and topoisomerase I inhibition for this novel class of inhibitor that lacks the structural lability of the camptothecins.

[1] Y. Pommier, (1998), Biochimie, 80, 255.

**P229**

**INTERACTION BETWEEN SN-38 AND 5-FLUOROURACIL (5-FU) IN RENAL CANCER CELL LINES**

V Sayer, N Darvill, S Joel, T Oliver, J Shamash. Dept Medical Oncology, St Bartholomew's Hospital, London.

Although renal tumours generally have low sensitivity to conventional cytotoxic agents, 2 of the most active drugs are 5-FU and Irinotecan. Recent data suggests that 5-FU and SN-38, the active metabolite of Irinotecan, are synergistic in colorectal cancer cell lines when SN-38 is followed by 5-FU (Pavillard et al, Biochem Pharm 1998, 56(10):1315-22). We have therefore investigated the activity of 5-FU/SN-38 combinations in a panel of renal cancer cell lines (RCC 39, RCC42, RCC45, kindly provided by Dr Terry Rabbits, MRC, Cambridge), focussing particularly on the effect of sequence of exposure. Firstly, the $EC_{50}$ (% viability, (%V)) for each drug in each cell

© 2002 Cancer Research UK

